# Editorial: Early neural processing of musical melodies

**DOI:** 10.3389/fnhum.2022.1109500

**Published:** 2022-12-12

**Authors:** André Rupp, Bernhard Englitz, Emili Balaguer-Ballester, Martin Andermann

**Affiliations:** ^1^Section of Biomagnetism, Department of Neurology, Heidelberg University Hospital, Heidelberg, Germany; ^2^Donders Institute for Brain, Cognition and Behaviour, Radboud University, Nijmegen, Netherlands; ^3^Faculty of Science and Technology, Bournemouth University, Bournemouth, United Kingdom

**Keywords:** MEG, melody processing, N100m, auditory cortex, music

In recent years, various endeavors have been made in neuroscience, computational modeling and psychoacoustics to better understand how musical melodies are represented in the human auditory system. Some of these investigations have dealt with organizational (i.e., adaptive and/or hierarchical) structure, while others have focused more on the role of individual characteristics like, e.g., the listener's musicality. With regards to early neurophysiological processing, however, an integrative perspective on melody is only about to evolve. The purpose of our Frontiers Research Topic “*Early neural processing of musical melodies*” is, therefore, to collect studies that analyze *early* cortical melody processing, as a pre-requisite for higher-order, holistic representations.

The current Research Topic comprises four works which target different but closely related issues in the early physiological processing of melodic information at the level of auditory cortex. Three of these works report data from magnetoencephalography experiments (MEG); they tackle spatio-temporal differences in the activity evoked by sequences with fixed vs. varying pitch (Taddeo et al.), the integration of deviant representations in melodies and the role of musicality (Hansen et al.), and attentional effects in the cortical tracking of voice pitch (Brodbeck and Simon). The fourth paper is a perspective article (Gande); it presents a comprehensive view where neural melody processing is discussed in light of overarching musical cognition and performance.

Taddeo et al. investigate the neuromagnetic response to brief melodic contours and compare it with the activity elicited by fixed-pitch sequences. The source activity is projected onto a finely parceled anatomical atlas; in line with earlier fMRI findings, there appears an anatomical and functional gradient in the early cortical processing where posterior activity reflects pitch sequence onset and anterior activity reflects the subsequent notes, including the difference between sequences with fixed pitch and melodic contours. The spatial separation can be interpreted in light of the dual-stream hypothesis which suggests that melody processing is attributed to an anterior stream while spatial processing occurs with the posterior stream.

The study of Hansen et al. builds on a paradigm that is different from Taddeo et al.'s work; they report a complex mismatch study in which the deviant responses to varying acoustic features in musical melodies are jointly analyzed, taking into account the musical expertise of the participants. Compared with non-musicians, musicians show a greater degree of subadditivity in their combined mismatch response to one or more deviant sound features; in contrast, no differences between groups are visible when the same stimuli are presented in “classic” oddball paradigms. The authors interpret this result pattern as a corroboration of the integrated processing hypothesis according to which overlapping resources in neural processing enable efficient complex representations of relevant structures in sound; moreover, in light of this notion, the musicality-related differences in this study are of particular importance.

Pitch contour tracking is a crucial pre-requisite not only for the processing of musical melodies, but also in the case of speech prosody. This forms the starting point for the experiment of Brodbeck and Simon where listeners track the voice pitches of competing speakers: In single-speaker conditions, pitch contour and pitch salience are continuously represented in the activity of the superior temporal gyrus within the N100m time window; on the other hand, when listeners hear a mixture of two speakers, voice pitch tracking follows the fundamental frequency of the attended speaker while the respective information of the unattended speaker is not represented. This result pattern demonstrates the important role of attentional processes even in early cortical representations of pitch; moreover, it supports the notion that speaker segregation in cocktail party scenarios occurs post-attentively.

The three empirical studies within our Research Topic are complemented by the perspective article of Gande which takes a multidimensional, interdisciplinary view on neural melody processing and well beyond. Gande refers to the well-established Type I/Type II dual processing model, describes its balance for different musical demands, and discusses its implications in the context of non-improvising musical performance. The work touches on a plethora of moderator variables that all shape the neural representation of music, ranging from melody processing to aspects of creativity and interpretation; in this vein, it bridges the gap between music as a structured auditory scene and musical experience and practice in a broader sense, also including philosophical aspects.

Taken together, the above-described works nicely demonstrate both the manifold experimental approaches to neurophysiological data and the need to closely relate the respective findings to real-world musical practice. We hope that the papers collected in this Research Topic will help to stimulate future research on the neural signature of musical melodies, especially with respect to the numerous factors that impact its early correlates in the auditory cortex and the functional interplay with higher, but also with subcortical processing stages.

## Author contributions

All authors contributed to this manuscript and approved its final version.

